# COVID-19 vaccines reduce the risk of SARS-CoV-2 reinfection and hospitalization: Meta-analysis

**DOI:** 10.3389/fmed.2022.1023507

**Published:** 2022-11-09

**Authors:** Maria Elena Flacco, Cecilia Acuti Martellucci, Valentina Baccolini, Corrado De Vito, Erika Renzi, Paolo Villari, Lamberto Manzoli

**Affiliations:** ^1^Department of Environmental and Prevention Sciences, University of Ferrara, Ferrara, Italy; ^2^Department of Public Health and Infectious Diseases, Sapienza University of Rome, Rome, Italy; ^3^Department of Medical and Surgical Sciences, University of Bologna, Bologna, Italy

**Keywords:** SARS-CoV-2, COVID-19, vaccination, meta-analysis, Omicron (B.1.1.529), reinfection

## Abstract

The addictive protection against SARS-CoV-2 reinfection conferred by vaccination, as compared to natural immunity alone, remains to be quantified. We thus carried out a meta-analysis to summarize the existing evidence on the association between SARS-CoV-2 vaccination and the risk of reinfection and disease. We searched MedLine, Scopus and preprint repositories up to July 31, 2022, to retrieve cohort or case-control studies comparing the risk of SARS-CoV-2 reinfection or severe/critical COVID-19 among vaccinated vs. unvaccinated subjects, recovered from a primary episode. Data were combined using a generic inverse-variance approach. Eighteen studies, enrolling 18,132,192 individuals, were included. As compared to the unvaccinated, vaccinated subjects showed a significantly lower likelihood of reinfection (summary Odds Ratio—OR: 0.47; 95% CI: 0.42–0.54). Notably, the results did not change up to 12 months of follow-up, by number of vaccine doses, in studies that adjusted for potential confounders, adopting different reinfection definitions, and with different predominant strains. Once reinfected, vaccinated subjects were also significantly less likely to develop a severe disease (OR: 0.45; 95% CI: 0.38–0.54). Although further studies on the long-term persistence of protection, under the challenge of the new circulating variants, are clearly needed, the present meta-analysis provides solid evidence of a stronger protection of hybrid vs. natural immunity, which may persist during Omicron waves and up to 12 months.

## Introduction

Clarifying the frequency and predictors of SARS-CoV-2 reinfections is crucial to determine the course of the pandemic, and to optimize restriction and vaccination policies (1–3). Solid evidence is currently available on the frequency of reinfections after the emergence of the Omicron variant: a recent proportion meta-analysis including 15 million subjects recovered from a first infection estimated an overall reinfection rate of 3.3% in the first 3 months of Omicron predominance, likely increasing (2). However, the potential addictive protection conferred by hybrid immunity, generated by the combination of prior infection and vaccination, as compared to the sole natural immunity, remains to be fully disclosed (4, 5). A few population-based studies suggested that reinfection is less likely in vaccinated vs. unvaccinated subjects, but the magnitude of the association varied across studies, which differed for patients’ characteristics, exposure risk, type of SARS-CoV-2 vaccine received, definition of reinfection adopted, and extent of measured confounding (4, 6–8). In a recent meta-analysis, the overall reinfection rate among vaccinated subjects was quantified to be as low as 0.32%, as compared to 0.74% among previously infected, unvaccinated individuals, but these estimates were obtained from raw, unadjusted data (2). Additionally, only limited data are available on the time course of natural and hybrid immunity (9), and the extent of its waning, particularly due to Omicron infections, is not yet well characterized (4, 9).

We carried out a meta-analysis to summarize the existing evidence from adjusted analyses on the association between SARS-CoV-2 vaccination and reinfection, in subjects who recovered from a first episode of SARS-CoV-2 infection.

## Methods

### Bibliographic search, data extraction and quality assessment

The reporting of this meta-analysis was guided by the standards of the Preferred Reporting Items for Systematic Review and Meta-Analysis (PRISMA) 2020 Statement (10). We searched MedLine and Scopus databases, up to July 31, 2022, for studies evaluating the risk of SARS-CoV-2 reinfection (either asymptomatic or symptomatic and requiring hospital admission) among vaccinated subjects of all ages (with hybrid immunity resulting from a combination of natural and vaccine immunization), vs. unvaccinated subjects (with natural immunity only). Vaccinated subjects were defined as those receiving ≥ 1 dose of the COVID-19 vaccines currently approved ≥ 14 days before the reinfection. The following search strategy was adopted, without language restrictions: (coronavirus* or coronovirus* or coronavirinae* or Coronavirus* or Coronovirus* or Wuhan* or Hubei* or Huanan or “2019-nCoV” or 2019nCoV or nCoV2019 or “nCoV-2019” or “COVID-19” or COVID19 or “WN-CoV” or WNCoV or “HCoV-19” or HCoV19 or CoV or “2019 novel*” or Ncov or “n-cov” or “SARS-CoV-2” or “SARSCoV-2” or “SARSCoV2” or “SARS-CoV2” or SARSCov19 or “SARS-Cov19” or “SARSCov-19” or “SARS-Cov-19” or Ncovor or Ncorona* or Ncorono* or NcovWuhan* or NcovHubei* or NcovChina* or NcovChinese*) AND (reinfection* or re-infection* or second episode or recurrence* or recrudescence* or relapse* or RCOVID19) (2). The reference lists of reviews and retrieved articles was also screened, for additional pertinent papers (11). Given that several relevant clinical databases have been shared in public preprint repositories in the context of a public health emergency, we also searched for potential studies among those submitted in medRxiv.org. In case of re-analyses published from the same cohort, we extracted the data of the publication with the longer follow-up or, if the length of follow-up was identical, with the largest sample size.

Inclusion criteria were: (a) cohort or case-control design; (b) laboratory confirmation of SARS-CoV-2 initial episode through a positive reverse-transcriptase polymerase chain reaction (RT-PCR) test, and/or an initial positive serology investigated with the use of an anti-trimeric spike IgG enzyme-linked immunosorbent assay (ELISA) (12); (c) data available to compare SARS-CoV-2 reinfection by vaccination status in subjects who recovered from a primary infection; (d) explicit reinfection definition criteria. In accordance with CDC (12), a reinfection was defined by the presence of:

(a) two positive PCR samples detected ≥ 45 days apart with ≥ 1 negative RT-PCR test collected between the first and second episode (13), and/or confirmation of infection with two different phylogenetic strains by viral genomic sequencing;

(b) two positive PCR samples detected ≥ 45 days apart in subjects with a symptomatic second episode or in close contact with a laboratory-confirmed COVID-19 case (12);

(c) a positive PCR test ≥ 45 days after the first positive serology (detection of anti-S1 domain of spike protein IgG antibodies using an enzyme-linked immunosorbent assay—ELISA) (12, 14).

Each included article was independently evaluated by 2 reviewers (MEF, CAM), who extracted the main study characteristics and measures of effect. In case of discrepancies in data extraction, a third author was contacted (LM), and consensus achieved through discussion.

Individual study quality was evaluated using an adapted version of the Newcastle Ottawa Quality Assessment Scale, assessing the comparability across groups for confounding factors, the appropriateness of outcome assessment, length of follow-up and missing data handling and reporting (15).

### Data analysis

The units of the meta-analysis were single comparisons of vaccinated vs. unvaccinated subjects in predicting (a) SARS-CoV-2 reinfection; (b) severe COVID-19 disease—requiring hospital admission with no use of an intensive care unit; (c) critical/lethal COVID-19 disease—requiring admission in an intensive care unit and/or causing death (2). The likelihood of each outcome was assessed: (a) using ≥ 45 days as the minimum time-lag between two positive episodes; (b) adopting a more stringent time-lag of 90 days (2); (c) including only studies with adjusted estimates. When data were available, we also performed several additional meta-analyses stratified by: (d) number of vaccine doses (“fully vaccinated” subjects—those receiving ≥ 2 doses of mRNA-1273, BNT162b2, ChAdOx1 nCoV-19, BBV152, BBIBP-CorV, Gam-COVID-Vac, CoronaVac, or 1 dose of JNJ-78436735 ≥ 14 days before reinfection—or “partially vaccinated” subjects—those receiving 1 dose of mRNA-1273, BNT162b2, ChAdOx1 nCoV-19, BBV152, BBIBP-CorV, Gam-COVID-Vac, or CoronaVac ≥ 14 days before reinfection—vs. unvaccinated) (13). When data were available, we also extracted separate estimates for those who received 3 doses of mRNA-1273, BNT162b2, ChAdOx1 nCoV-19, BBV152, BBIBP-CorV, Gam-COVID-Vac, or CoronaVac vaccines (“boosted subjects”); (e) time between first episode and reinfection (<6 vs. ≥ 6 months); (f) dominant viral strain (Delta or Omicron); (g) exposure risk (healthcare workers or general population); (h) study design (cohort or case-control).

Data were combined using a random-effect generic inverse variance approach (16, 17), in order to account for between-study heterogeneity (18). If a study reported the results of different multivariable models, the most stringently controlled estimates (those from the model adjusting for more factors) were extracted. If different models controlled for the same number of covariates, the model containing the most clinically meaningful covariates was used for the analysis (19). When a study only reported separate estimates by vaccine dose, the overall estimate of risk was computed from the separate relative risks using the fixed-effect model for generic inverse-variance outcomes (19).

Between-study heterogeneity was quantified using the I^2^ statistic. Potential publication bias was assessed graphically, using funnel plots [displaying the Odds Ratios—ORs from individual comparisons vs. their precision (1/SE)], and formally, using Egger’s regression asymmetry test (16).

All meta-analyses were performed using RevMan software, version 5.3 [The Cochrane Collaboration, (20)].

## Results

Of the 3,470 papers initially retrieved, seven case-control (4, 21–26) and 11 cohort studies (6–8, 27–34) were included in the analyses (Supplementary Figure 1 and Supplementary Table 1). Three studies contributed with two dataset (24, 27, 31), as the same publication provided separate data for healthcare workers and the general population (31), and for Delta and Omicron waves (24, 27): this led to a total of 21 datasets that were included in the analyses (Table 1).

**TABLE 1:** Characteristics of the included studies.

No.	References	Journal	Country	Design	Population	% vacc.	Mean age (SD)	Mean f-up (days)	Dominant strain	Reinfection definition and time-lag	Raw data^a^	Covariates
1	Bager et al. a1 ^b^ (27)	Lancet Infect Dis	Denmark	Cohort	General	65.8	31.0 (27.4)	120	Delta	2 PCR + > 60 days	783/80 426 vs. 1103/69,885	Raw data extracted
2	Bager et al. a2 ^b^ (27)	Lancet Infect Dis	Denmark	Cohort	General	81.2	29.0 (18.5)	120	Omicron	2 PCR + > 60 days	1520/31 403 vs. 622/7266	Raw data extracted
3	Cavanaugh et al. (21)	MMWR	USA	Case-control	General	20.3	NR	NR	NR	PCR + /Ag test May–Jun21 (1st episode: Mar-ec 20)	67/275 vs. 179/463	Age, gender, time from 1st infection
4	Cerqueira-Silva et al. (26)	Lancet Infect Dis	USA	Case-control	General	35.5	36.0 (11.1)	60	Gamma	2 PCR + > 90 days	6584/59,064 vs. 14 566/97 856	Comorb, time from 1st infection, severity of 1st infection
5	Eythorsson et al. (6)	JAMA Netw Open	Iceland	Cohort	General	25.5	34.0 (19.0)	287	Omicron	2 PCR + > 60 days	320/2938 vs. 1007/8598	Age, gender, time from 1st infection
6	Flacco et al. (28)	Front PublicHealth	Italy	Cohort	General	43.5	41.6 (21.9)	277	Omicron	2 PCR + ≥ 45 days (≥ 1 PCR−)	386/88,576 vs. 343/30,690	Age, gender, comorb, severity of 1st infection
7	Hall et al. (29)	Lancet	UK	Cohort	HCW	47.5	45.6 (14.2)	275	NR	2 PCR + ≥ 90 days + serology/genomic)	NR	Age, gender, ethnicity, time from 1st infection, workplace, contact frequency
8	Hammerman et al. (7)	New Engl J Med	Israel	Cohort	General	56.0	39.3 (17.1)	270	Delta	2 PCR + > 90 days	354/83,356 vs. 2,168/65,676	Age, gender, comorb. ethnicity, socio-economic status
9	Jang et al. (30)	J Med Virol	Korea	Cohort	General	76.1	NR	242	Omicron	2 PCR + ≥ 45 days	19,943/12,270,241 vs. 19,513/3,638,932	Age, gender, strain immunologic status
10	Levin-Rector et al. (22)	Clin Infect Dis	USA	Case-control	General	54.4	NR	NR	Delta	2 PCR + > 90 days	965/5,228 vs. 1,436/4,376	Age, gender, time from 1st infection
11	Lewis et al. a1 ^c^ (31)	JAMA Netw Open	USA	Cohort	General	51.2	35.0 (20.7)	225	Delta	2 PCR + > 90 days	298/52,683 vs. 1,105/41,833	Age, gender, time from and severity of 1st infection
12	Lewis et al. a2 ^c^ (31)	JAMA Netw Open	USA	Cohort	HCW	66.3	41.0 (17.0)	225	Delta	2 PCR + > 90 days	47/2,131 vs. 227/746	Age, gender, time from and severity of 1st infection
13	Malhotra et al. (32)	JAMA Netw Open	India	Cohort	HCW	75.3	36.6 (10.3)	233	Delta	2 PCR + ≥ 90 days	56/1,445 vs. 60/472	Age, gender, work category
14	Medic et al. (4)	Lancet Reg Health	Serbia	Case-control	General	46.2	45.9 (18.7)	340	Omicron	Rapid Ag test or 2 PCR + ≥ 90 days	3,404/10,220 vs. 3,815/11,417	Age, gender, comorb., time from 1st infection
15	Murugesan et al. (33)	PloS One	India	Cohort	HCW	76.9	33.7 (10.9)	259	Delta	2 PCR + ≥ 90 days	12/791 vs. 16/658	Raw data extracted
16	Nisha et al. (34)	J Fam Commun Med	India	Cohort	HCW	36.3	30.3 (10.5)	270	NR	2 PCR + > 90 days (≥ 1 PCR−)	103/1,684 vs. 24/225	Age, gender, comorb, work category
17	Nordstrom et al. (8)	Lancet Infect Dis	Sweden	Cohort	General	50.0	38.8 (17.9)	60	Delta	PCR + Dec 20-Oct 21 (1st episode before 24 May 21)	1,077/765,064 vs. 2,470/765,064	Age, gender, comorb., time from 1st infection, marital status, work category
18	Nunes et al. (23)	Vaccines	South Africa	Case-control	HCW	80.0	37.4 (9.2)	NR	Omicron	2 PCR + > 90 days	43/116 vs. 9/23	Study site
19	Plumb et al. a1 ^b^ (24)	MMWR	USA	Case-control	General	48,4	NR	NR	Delta	2 PCR + > 90 days	487/2,183 vs. 950/2,418	Age, gender, race, time from 1st infection
20	Plumb et al. a2 ^b^ (24)	MMWR	USA	Case-control	General	48,4	NR	NR	Omicron	3 PCR + > 90 days	971/3,442 vs. 1,353/3,456	Age, gender, race, time from 1st infection
21	Spicer et al. (25)	J Pediatric	USA	Case-control	General	20.5	15.1 (1.7)	246	Delta	2 PCR + > 90 days	20/855 vs. 342/3,307	Raw data extracted

HCW, healthcare workers; SD, standard deviation; % vacc., % of vaccinated individuals; Comorb., Comorbidities; PCR, oro-nasopharyngeal swabs tested through reverse transcription polymerase chain reaction; PCR +, Positive RT-PCR; PCR−, Intermediate negative RT-PCR between two positive tests; Ag, Antigen; NR, Not reported.

^a^Raw data: Number of vaccinated, reinfected subjects/Total number of vaccinated subjects vs. Number of unvaccinated, reinfected subjects/Total number of unvaccinated subjects.

^b^Same publication providing separate data for Delta and Omicron waves.

^c^Same publication providing separate data for general population and healthcare workers.

Six studies were carried out in Europe (4, 6, 8, 24, 27–29), six in the USA (21, 22, 24–26, 31), five in Asia (7, 30, 32–34) and one in South Africa (23). Thirteen studies evaluated the general population (4, 6–8, 21, 22, 24–28, 30, 31), and six assessed the healthcare workers (23, 29, 31–34). In most studies, the analyses were adjusted for age, gender, and comorbidities, as a minimum set of potential confounders of the association between vaccination status and reinfections (4, 6–8, 21, 23, 26, 28–32, 34).

The mean age of the participants ranged from 15 to 46 years, and the mean follow-up ranged from a minimum of 60 up to 340 days. In 13 studies (4, 7, 21–26, 29, 31–34) the minimum time-lag between infection and reinfection was set at 90 days, and only three (28, 29, 34) strictly followed the CDC criteria to define a reinfection (≥1 intermediate negative PCR and/or viral genomic sequencing) (12). Most reinfections were reported during the Delta (7, 8, 22, 24, 25, 27, 31–33) and the Omicron waves (4, 6, 23, 24, 27, 28, 30).

The methodological characteristics of the included studies are summarized in Table 2: the selection of the cohort of patients, the ascertainment of the exposure, and the evaluation of the comparability of subjects were adequate in all studies, while 15 out of 18 adequately addressed the items pertaining to outcome assessment and follow-up (length and missing data).

**TABLE 2:** Methodological quality of the included studies according to the Newcastle Ottawa Scale.

References	Selection	Comparability	Outcome
	(Max. score 4)	(Max. score 2)	(Max. score 3)
Bager et al. (27)	4	2	3
Cavanaugh et al. (21)	4	2	3
Cerqueira-Silva et al. (26)	4	2	3
Eythorsson et al. (6)	4	2	3
Flacco et al. (28)	4	2	3
Hall et al. (29)	4	2	3
Hammerman et al. (7)	4	2	3
Jang et al. (30)	4	2	3
Levin-Rector et al. (22)	4	2	2
Lewis et al. (31)	4	2	3
Malhotra et al. (32)	3	2	3
Medic et al. (4)	4	2	3
Murugesan et al. (33)	4	2	3
Nisha et al. (34)	4	2	3
Nordstrom et al. (8)	4	2	2
Nunes et al. (23)	3	2	2
Plumb et al. (24)	4	2	3
Spicer et al. (25)	4	2	2

**FIGURE 1:**
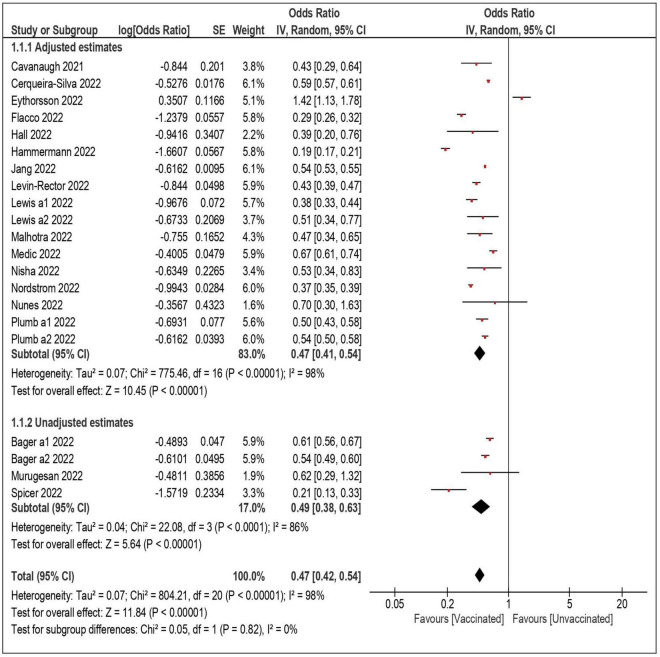
Risk of SARS-CoV-2 reinfection among vaccinated vs. unvaccinated subjects.

Twenty-one datasets including a total of 18,132,192 individuals were included in the overall meta-analysis comparing the risk of SARS-CoV-2 reinfection in vaccinated vs. unvaccinated subjects (Table 3) (4, 6–8, 21–34). In 20 out of 21 datasets, the vaccinated subjects were significantly less likely to be reinfected, with a summary OR of 0.47 (95% confidence interval—CI – 0.42–0.54) (Figure 1). When the only study reporting a significantly higher risk among vaccinated subjects (and no data on underlying comorbidities) was excluded (6), the estimates were virtually identical (OR: 0.45; 95% CI: 0.39–0.50). Also, the results did not substantially change after the exclusion of the three studies with unadjusted estimates (OR: 0.47; 95% CI: 0.39–0.56) (25, 27, 33), and when only the 17 datasets with a more conservative time-lag of 90 days were considered (OR: 0.47; 95% CI: 0.41–0.54) (4, 7, 21, 23, 24, 26, 29, 31–34).

**TABLE 3:** Risk of SARS-CoV-2 reinfection and severe/critical COVID-19 among vaccinated vs. unvaccinated subjects, overall, and stratified by definition of reinfection, number of vaccine doses, length of follow-up, predominant strain, study design and risk exposure.

		Pooled estimates			Raw data^b^			
			
*Analyses*	N. datasets	OR (95% CI)	*P*-value	*I*^2^, %	No. of events	Vaccinated subjects	No. of events	Unvaccinated subjects
	(total sample)^a^							
**SARS-CoV-2 reinfection—all studies** (4, 6, 8, 21–34)	21(18,132,192)	0.47(0.42−0.54)	<0.001	98	37,440	13,462,121	134,598	4,670,071
- Adjusted estimates only (4, 6, 8, 21–24, 26, 28–32, 34)	17(17,937,601)	0.47(0.41−0.54)	<0.001	98	35,105	13,348,646	132,525	4,588,955
**1. Time-lag ≥ 90 days ^c^** (4, 7, 21–26, 29, 31–34)	15(373,109)	0.44(0.36−0.54)	<0.001	97	13,411	223,473	109,540	149,636
- Adjusted estimates only (4, 21–24, 26, 29, 31, 32, 34)	13(367,498)	0.46(0.37−0.56)	<0.001	97	13,379	221,827	109,182	145,671
**2. Number of vaccine doses**: ^d^								
- Partially vaccinated subjects (4, 8, 23, 24, 26, 28, 30–32)	11(5,248,720)	0.58(0.44−0.77)	0.004	98	5,820	729,103	127,701	4,509,617
- Fully vaccinated subjects (4, 8, 21–24, 26, 28, 30–32)	13(17,036,021)	0.45(0.40−0.50)	<0.001	95	28,508	12,521,565	129,316	4,514,456
- Boosted subjects (3 doses) (4, 24, 30)	4(11,365,430)	0.46(0.29−0.73)	0.001	99	1,675	7,709,207	25,631	3,656,223
**3. Length of follow-up:**								
- <6 months (< 120 days)—all studies (8, 26, 27)	4(1,876,028)	0.52(0.40−0.67)	<0.001	99	9,964	935,957	18,761	940,071
- Adjusted estimates only (8, 26)	2(1,603,758)	0.47(0.30−0.74)	0.001	99	7,661	824,128	17,036	862,920
- ≥6 months (≥ 120 days)—all studies (4, 6, 7, 25, 28–34)	12(16,317,474)	0.45(0.34−0.59)	0.005	98	24,943	12,514,920	28,620	3,802,554
- Studies with adjusted estimates only (4, 6, 7, 28–32, 34)	10(16,311,863)	0.47(0.35−0.63)	0.05	99	24,911	12,513,274	28,262	3,798,589
**4. Predominant viral strain:**								
- Delta variant (B.1.617.2)—all studies (8, 22, 24, 25, 27, 31–33)	10(1,948,597)	0.40(0.31−0.50)	<0.001	97	4,099	994,162	9,877	954,435
- Adjusted estimates only (8, 22, 24, 31, 32)	7(1,792,675)	0.38(0.30−0.49)	<0.001	96	3,284	912,090	8,416	880,585
- Omicron variant (B.1.1.529)—all studies (4, 6, 23, 24, 27, 28, 30)	7(16,107,318)	0.58(0.48−0.70)	<0.001	97	26,587	12,406,936	26,662	3,700,382
- Adjusted estimates only (4, 6, 23, 24, 28, 30)	6(15,951,396)	0.59(0.48−0.73)	<0.001	96	25,772	12,324,864	25,01	3,626,532
**5. Risk of exposure:**								
- General population—all studies (4, 6, 8, 21, 22, 24–28, 30, 31)	15(18,123,901)	0.47(0.41−0.53)	<0.001	98	37,179	13,455,954	134,262	4,667,947
- Adjusted estimates only (4, 6, 8, 21, 22, 24, 26, 28, 30, 31)	11(17,930,759)	0.46(0.37−0.55)	<0.001	98	34,856	13,343,270	132,195	4,587,489
- Healthcare workers—all studies (23, 29, 31–34)	6(8,291)	0.50(0.41−0.61)	<0.001	0	261	6,167	336	2,124
- Adjusted estimates only (23, 29, 31, 32, 34)	5(6,842)	0.49(0.40−0.61)	<0.001	0	249	5,376	320	1,466
**6. Study design:**								
- Cohort—all studies (6, 8, 25, 27–34)	14(18,014,945)	0.44(0.36−0.54)	<0.001	98	24,919	13,381,593	29,000	4,633,352
- Adjusted estimates only (6, 8, 28–32, 34)	10(17,820,354)	0.44(0.33−0.57)	<0.001	98	22,584	13,268,118	26,917	4,552,236
- Case-control—all studies (4, 21–24, 26)	7(117,247)	0.54(0.48−0.61)	<0.001	89	12,521	80,528	105,598	36,719
**Severe or critical/lethal COVID-19 ^e^** (8, 22, 24, 26, 29, 32)	7(2,312,703)	0.45(0.38−0.54)	<0.001	91	1,411	1,536,917	2,657	775,786
**1. Number of vaccine doses**: ^d^								
- Partially vaccinated subjects (8, 24, 26, 32)	5(982,721)	0.35(0.21−0.60)	0.02	91	474	48,4471	3,693	498,250
- Fully vaccinated subjects (8, 22, 24, 26, 32)	6(597,193)	0.34(0.24−0.49)	<0.001	93	1,629	296,197	3,620	300,996

^a^Three studies (24, 27, 31) contributed with more than one dataset, thus the number of references does not always match the number of datasets included in each analysis (see “Results” for further details).

^b^Number of events/Total number of previously infected and vaccinated subjects vs. Number of events/Total number of previously infected and unvaccinated subjects.

^c^The risk of SARS-CoV-2 reinfection was computed: (1) using ≥ 45 days as the minimum time-lag between two positive episodes; (2) adopting a more stringent time-lag of 90 days (see Methods for further details).

^d^Partially vaccinated subjects: 1 dose of mRNA-1273, BNT162b2, ChAdOx1 nCoV-19, BBV152, BBIBP-CorV, Gam-COVID-Vac, or CoronaVac ≥ 14 days before reinfection; fully vaccinated subjects: ≥ 2 doses of mRNA-1273, BNT162b2, ChAdOx1 nCoV-19, BBV152, BBIBP-CorV, Gam-COVID-Vac or CoronaVac, or 1 dose of JNJ-78436735 ≥ 14 days before reinfection; boosted subjects: 3 doses of mRNA-1273, BNT162b2, ChAdOx1 nCoV-19, BBV152, BBIBP-CorV, Gam-COVID-Vac, or CoronaVac vaccines.

^e^Severe COVID-19: disease requiring hospital admission with no use of an intensive care unit; critical/lethal COVID-19: disease requiring admission in an intensive care unit and/or causing death. OR, Odds ratio; CI, confidence interval.

When the analyses were stratified by number of doses, the summary OR of reinfection was lower among fully vaccinated than partially vaccinated subjects (summary OR 0.45 and 0.58, respectively). The confidence intervals, however, largely overlapped. In the analyses restricted to the subjects who received three doses (a booster dose), the summary OR was comparable to that of the fully vaccinated individuals (OR: 0.46; 95% CI: 0.29–0.73). As shown in Table 3, the association between vaccination and reinfection did not show a substantial variation by length of follow-up: the summary OR of the studies with a follow-up shorter than 6 months (OR: 0.52; 95% CI: 0.40–0.67) was comparable with the OR (0.45; 95% CI: 0.34–0.59) of the studies with a longer follow-up (up to 340 days).

The likelihood of a reinfection remained significantly lower among vaccinated subjects both in the studies that were carried out during Delta predominance (summary OR: 0.40; 95% CI: 0.31–0.50) (7, 8, 19, 22–24, 27–29) and during Omicron predominance (OR: 0.58; 95% CI: 0.48–0.60) (2, 4, 6, 23, 24, 27, 30). Again, in the analyses stratified by risk of exposure (general population or healthcare workers) and by study design (cohort or case-control) the likelihood of reinfection was comparably, significantly lower among vaccinated subjects, with summary ORs ranging from 0.44 to 0.54, and overlapping confidence intervals.

The Egger test was not significant (*p* = 0.3), and the funnel plot displaying the ORs of the individual comparisons vs. the logarithm of their SE (precision) did not show asymmetry, suggesting the absence of publication bias (Supplementary Figure 2).

**FIGURE 2:**
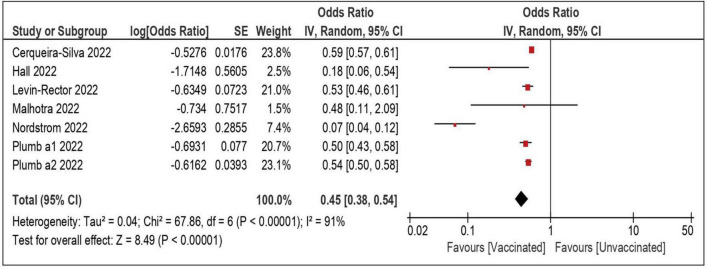
Risk of severe/lethal COVID-19 among vaccinated vs. unvaccinated subjects.

A total of seven datasets and 2,312,703 individuals provided specific data and were included in the meta-analysis comparing the risk of severe/lethal COVID-19 of the vaccinated vs. the unvaccinated subjects (8, 22, 24, 26, 29, 32). Compared with the unvaccinated, those receiving ≥ 1 dose were significantly less likely to develop a severe disease, once reinfected (OR: 0.45; 95% CI: 0.38–0.54—Table 3 and Figure 2). The risk remained comparably and significantly lower when only the subset of studies evaluating partial vaccination (OR: 0.35; 95% CI: 0.21–0.60) or those evaluating full vaccination (OR: 0.34; 95% CI: 0.24–0.49) vs. no vaccination, were included.

## Discussion

This meta-analysis, which included the data of more than 18 million previously infected and recovered subjects, has two main findings. First, as compared to natural immunity alone, the addition of vaccination approximately halved the odds of severe COVID-19, and the degree of protection was similar after a single or multiple doses. Second, the likelihood of reinfection was also reduced by approximately 50% among the vaccinated, and this finding was consistent in all stratified analyses, either extracting estimates adjusted for potential confounders or unadjusted, with follow-ups shorter or longer than 6 months, adopting different reinfection definitions, in both case-control and cohort studies, in the general population and healthcare workers alone, after a single or multiple vaccine doses, and irrespective of the predominant strain.

Preliminary evidence suggested that the protection conferred by hybrid immunity against reinfection was similar, or only marginally better, than the infection-induced or vaccine-induced immunity alone (5, 35). More recently, however, a proportion meta-analysis including 15 million previously infected and recovered individuals reported markedly lower rates of reinfection among vaccinated vs. unvaccinated subjects (0.32% vs. 0.74%), but these findings were based upon raw data and needed confirmation from adjusted estimates (2). The present meta-analysis expanded the previous and included 15 studies that adjusted the analyses for age, gender, comorbidities, and other potential confounders, providing solid evidence of a stronger protection of hybrid vs. natural immunity, which may persist during Omicron waves and up to 12 months.

Indeed, concerning the waning of the immunity, a 20% decline in the effectiveness of vaccination against first infection after 6 months was first showed in a meta-analysis including studies up to December 2021 (36). Then, evidence of waning protection both with hybrid and natural immunity 4 months after immunization was reported in some large prospective studies, which showed corresponding upward trends in reinfection absolute rates during time (5, 8, 9). In the present meta-analysis, the reinfection rates of the cohort studies with follow-up longer than 6 months were not distinctly higher (0.17 and 0.65 × 100 individuals in vaccinated and unvaccinated subjects, respectively), as compared to those with short follow-up (0.39 and 0.50 × 100 individuals in vaccinated and unvaccinated subjects, respectively). Additionally, we did not observe a substantial reduction of the protection when the follow-up lasted 6–11 months: pooling the 12 datasets with a longer follow-up, the odds of reinfection were approximately 50% lower among the vaccinated. Inevitably, this information remains preliminary, as it is based upon studies in which the follow-up lasted up to 12 months, and the use of viral genomic sequencing was uneven.

These findings may offer a contribution to help planning tailored immunization strategies for previously infected subjects: if, on one side, the marked increase in the absolute number of reinfections with time is concerning, the significantly lower relative risk still observed among vaccinated subjects may be reassuring, thus vaccinating also this population may definitely play a role to control the pandemic (4). In this scenario, the strong protective effect exerted by a single dose (if confirmed during longer follow-up and toward different strains) might be taken into account when designing tailored vaccination schedules directed to lower-priority groups (4, 5). It should be also considered, however, that the degree of additional protection specifically conferred by further boosters (three or more doses) still remains uncertain, as our stratified meta-analyses did not show a clear benefit of a 3- vs. a 2-dose schedule.

The second main finding of the present meta-analysis was the significant reduction of the risk of hospitalization due to severe COVID-19 that was observed among the vaccinated subjects, either receiving one or more doses. This was crucial, as the primary aim of COVID-19 vaccination is to reduce the pressure on the healthcare systems preventing severe disease and hospitalization (37). Unfortunately, however, most of the studies included in the meta-analyses of this outcome were carried out before the emergence of Omicron strain. Therefore, this finding requires confirmation from more recent data with longer follow-up, as the large increase in the number of reinfections during the Omicron wave, and in turn the consequences on the healthcare systems still needs to be carefully evaluated.

In the first phases of the pandemic, there was uncertainty on the criteria to define a reinfection, especially on the time interval between the first and second episodes, and most initial studies defined a reinfection as a new PCR test occurring ≥ 90 days after complete resolution of the first infection (4, 7, 21–26, 29, 31–34). However, the CDC later expanded the definition, including also the subjects with COVID-19-like symptoms and detection of SARS-CoV-2 RNA ≥ 45 days since first infection (12). In the present analysis, we did not find substantial differences when a 90-day or a 45-day cutoff was adopted, suggesting that a low proportion of reinfections was missed using the longer threshold. Indeed, a recent cohort study reported a mean time between the first and second infection of 349 days, with less than 15% of the reinfections occurring in the first 6 months since the first episode (28).

Some limitations must be considered when interpreting the present findings. First, most meta-analyses showed an intermediate-to-high level of heterogeneity. However, a certain degree of heterogeneity across studies was inevitable, given the large variation in terms of setting and baseline patients characteristics. Also, when the analyses were repeated adopting a fixed approach, none of the results substantially differed (except for CIs, which were typically tighter). Second, although most studies provided analyses at least adjusted for age, gender, and several underlying comorbidities, some extent of residual confounding cannot be completely ruled out, as for any observational study (38). Third, the risk of reinfection could have been overestimated in several of the included studies adopting less stringent criteria to define a reinfection (2). Conversely, if previously infected people tended to seek fewer testing due to their presumed acquired natural immunity, the reinfection rate could have been underestimated (4). A sensitivity analysis based upon the average number of PCR tests as a proxy of health-seeking behavior would have increased the precision of our estimates (2), but these data were unfortunately not available. Fourth, it might have been interesting to evaluate if the results differed according to the sequence of events, whether vaccination was administered before or after the first infection. Unfortunately, however, the exact timeline of events could be determined only in two studies (4, 31), in which all the infections occurred before the start of the vaccination campaign.

Acknowledging these caveats, this meta-analysis showed that, among the subjects that recovered from a first SARS-CoV-2 infection, vaccination was associated with a significant and substantial reduction of the risk of both reinfection and severe COVID-19. This finding was confirmed when the analyses were adjusted for potential confounders, up to 12 months of follow-up, and after any vaccine dose. Further studies on the long-term persistence of protection, and assessing the reinfection and hospitalization rates under the challenge of the new circulating variants, are strongly warranted.

## Data availability statement

The data presented in this study are available upon reasonable request from the corresponding author.

## Author contributions

MF and LM: concept and design and statistical analysis. MF, CA, VB, and ER: acquisition, analysis, or interpretation of data. MF, CA, and LM: drafting of the manuscript. CD, PV, and LM: critical revision of the manuscript for important intellectual content. PV and LM: supervision. LM: full access to all the data in the study and take responsibility for the integrity of the data and the accuracy of the data analysis. All authors contributed to the article and approved the submitted version.
