# Supplementary material for: COVID-19 vaccines reduce the risk of SARS-CoV-2 reinfection and hospitalization: Meta-analysis
Source: Front Med (Lausanne). 2022 Nov 9;9:1023507. doi: 10.3389/fmed.2022.1023507 (PMC9681813; doi:10.3389/fmed.2022.1023507)
Supplement: Supplementary file 1 [file Data_Sheet_1.pdf]

Supplementary Material

Supplementary Figure captions

Supplementary Figure 1. PRISMA flow diagram.

Supplementary Figure 2. Funnel plot and Egger test for publication bias for the main outcome (SARS-CoV-2 reinfection).

Supplementary Table 1. Excluded studies, with reasons for the exclusion.

n	First author	Journal	Reason(s) for exclusion
1	Abu-Raddad	JAMA 2021	No control group
2	Al Mahdi	Int J Inf Dis 2022	No control group
3	Al Otaiby	Int J Inf Dis 2022	Control group: uninfected subjects at baseline
4	Altarawneh	NEJM 2022	Control group: uninfected subjects at baseline
5	Castillo	Eurosurv 2022	Control group: uninfected subjects at baseline
6	Cocchio	Int J Environ Res Pub Health 2022	Data not extractable
7	Cohen	Lancet Inf Dis 2022	No data on reinfections
8	Dhumal	Int J Inf Dis 2022	Data not extractable
9	Gallais	eBio Med 2022	Control group: uninfected subjects at baseline
10	Goldberg	NEJM 2022	Data not extractable
11	Hall	NEJM 2022	Control group: uninfected subjects at baseline
12	Kim	Clin Inf Dis 2022	No data on reinfections
13	Kim	J Inf Pub Health 2022	Case-series
14	Leon	MMWR 2022	Data not extractable
15	Lewnard	medRXive 2022	No data on reinfections
16	Lind	medRXive 2022	No data on reinfections
17	Malhotra	Lancet Reg Health 2022	Unclear reinfection definition
18	Michlmayr	Lancet Reg Health 2022	No data on vaccination
19	Neejevan	Emerg Inf Dis	Data not extractable
20	Pampa-Espinosa	OFIDIS 2022	No data on vaccination
21		Public Health 2022	Data not extractable
22	Rahman	Sci Rep 2022	Data not extractable
23	Rosenberg	BMC Inf Dis 2022	No data on vaccination
24	Rothberg	Clin Inf Dis 2022	No data on comparison by vaccination status
25	Sheikh	Lancet Inf Dis 2022	No data on comparison by vaccination status
