# Supplementary figures and images for: COVID-19 vaccines reduce the risk of SARS-CoV-2 reinfection and hospitalization: Meta-analysis
Source: Front Med (Lausanne). 2022 Nov 9;9:1023507. doi: 10.3389/fmed.2022.1023507 (PMC9681813; doi:10.3389/fmed.2022.1023507)

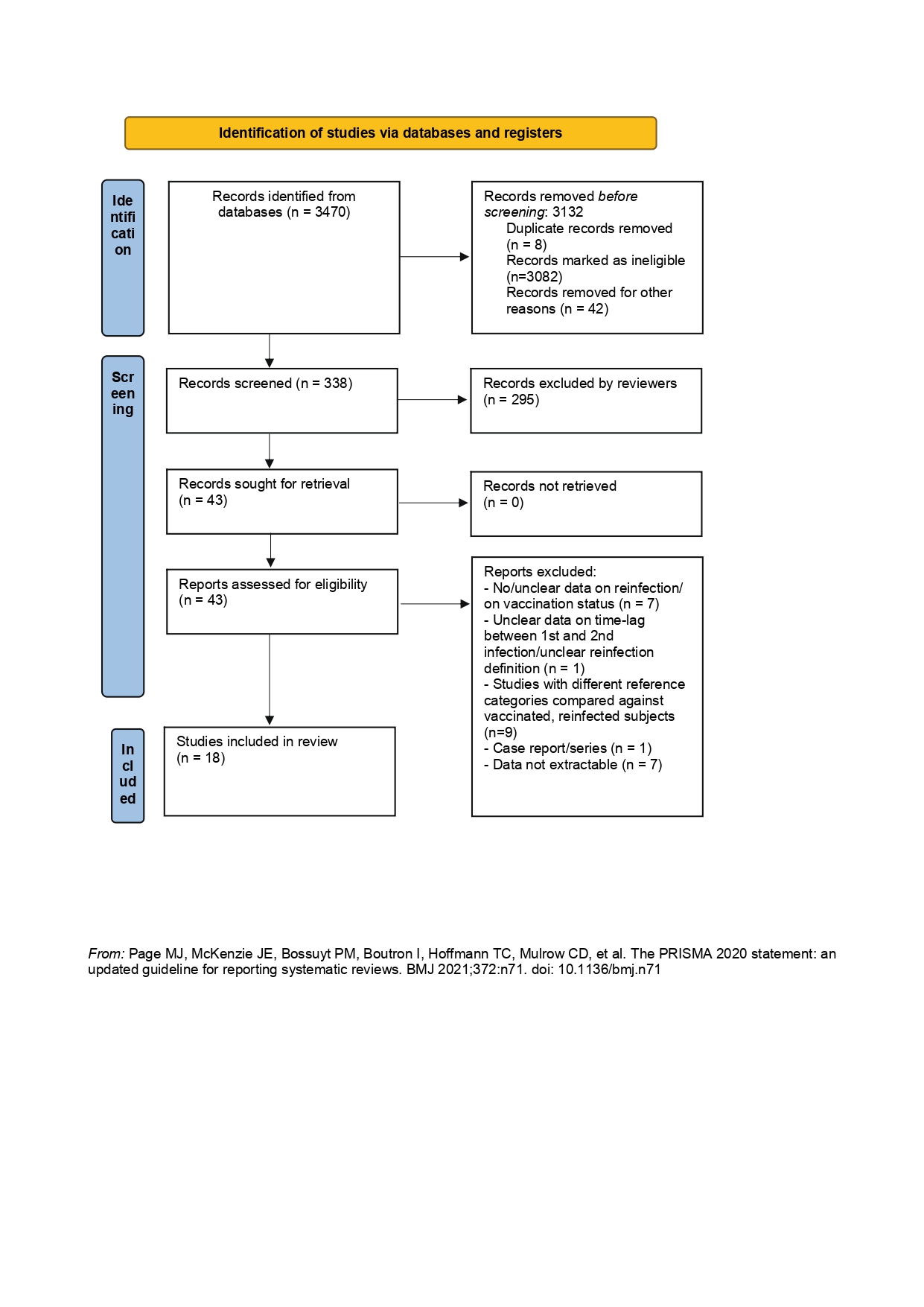

Supplement: Supplementary file 2 [file Image_1.JPEG]

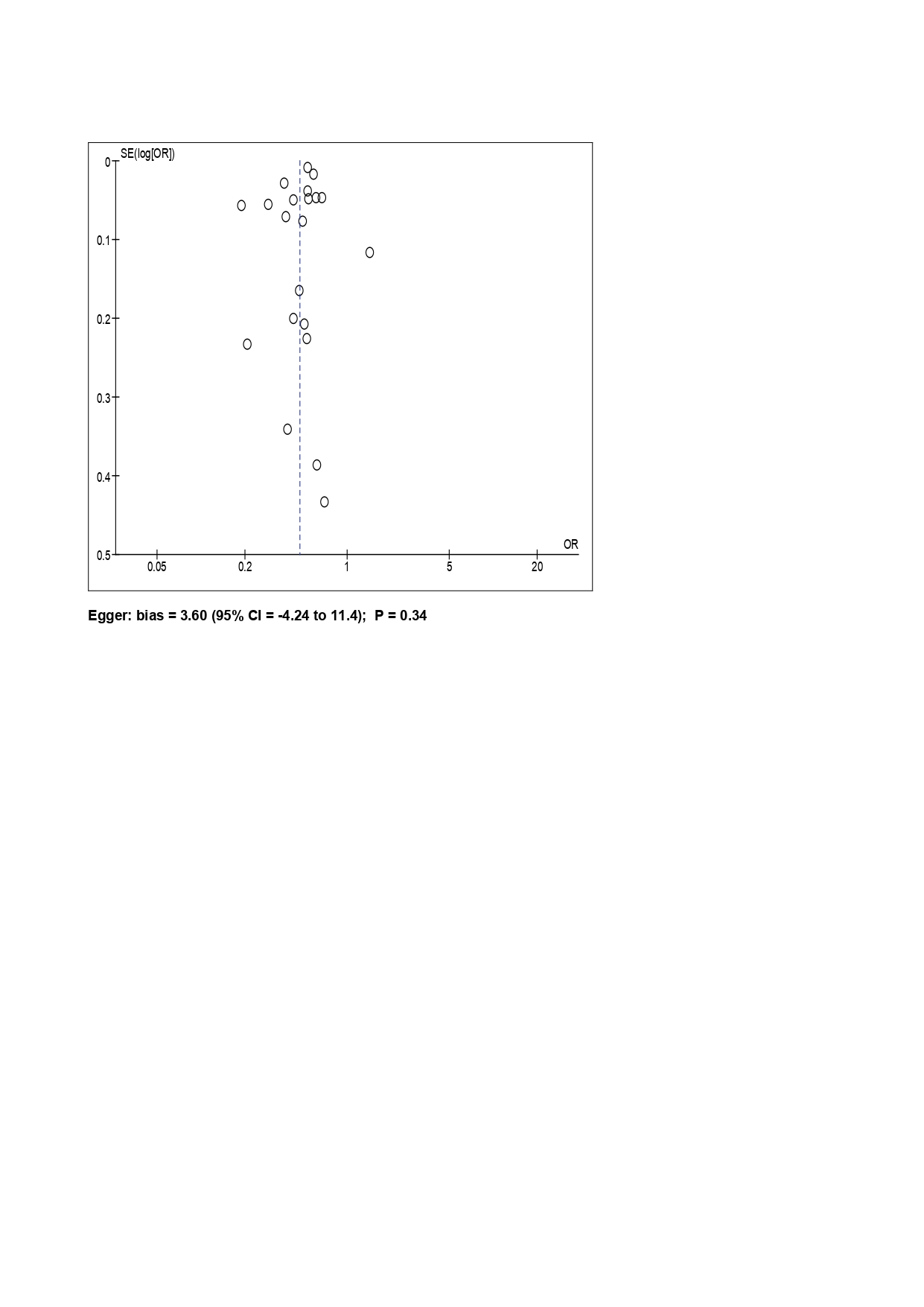

Supplement: Supplementary file 3 [file Image_2.JPEG]
